# P-887. Evaluation of ceftriaxone and cefotaxime as surrogate markers for oral second and third generation cephalosporins in *Escherichia coli* strains from blood

**DOI:** 10.1093/ofid/ofae631.1078

**Published:** 2025-01-29

**Authors:** Tho H Pham, Kyle Molina, Vanthida Huang

**Affiliations:** University of Arizona R Ken Coit College of Pharmacy, Phoenix, Arizona; Scripps Health, San Diego, California; Midwestern University, College of Pharmacy-Glendale Campus, Glendale, Arizona

## Abstract

**Background:**

Oral second- and third-generation cephalosporins are increasingly being utilized as oral transitional therapy for *Escherichia coli* bloodstream infections due to resistance or intolerance to other classes. However, most automated susceptibility systems only include cefazolin (CFZ), ceftriaxone (CRO), or cefotaxime (CTX). We aimed to evaluate the susceptibility correlation of CFZ, CRO, or CTX compared to oral second- and third-generation cephalosporins to determine a potential surrogate breakpoints.

**Figure:**
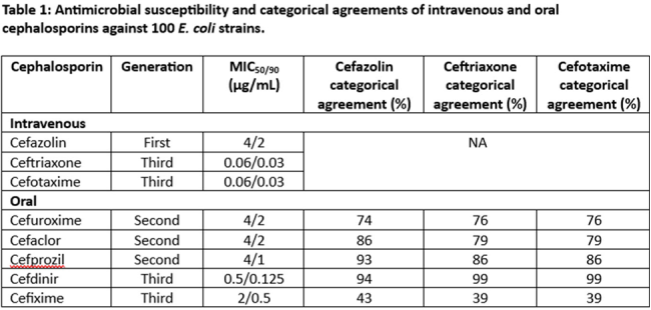

Antimicrobial susceptibility and categorical agreements of intravenous and oral cephalosporins against 100 E. coli strains

**Methods:**

We obtained 100 *E. coli* blood isolates from HonorHealth and Abrazo Health Systems in Phoenix, AZ from September 2021 to 2023. Broth microdilution was performed according to CLSI using commercially purchased CFZ, CRO, CTX, cefuroxime (CXM), cefaclor (CEC), cefprozil (CPR), cefdinir (CDR), and cefixime (CFM). *E. coli* ATCC 25922 was utilized as quality control. Categorical agreement and error rates were calculated for drug-drug combinations using CFZ, CRO, or CTX as the surrogate antibiotic for oral cephalosporins.

**Results:**

MIC_50/90_ values were reported in Table 1. With the recommended CFZ surrogate breakpoint of ≤ 16 µg/mL for oral cephalosporins, the categorical agreement rates were 86% for CEC and 93% for CPR. When applying CRO and CDR breakpoint of ≤ 1 µg/mL, highest categorical agreement rates of 99% were seen with CDR, followed by those of 86% with CPR. Compared to CFZ, CRO, and CXM resulted in higher false susceptible rates with CEC and CPR. However, if CRO and CTX MIC ≤0.06 µg/mL had been used, the categorical rates would have been above 95% with CEC and CPR. CFM susceptibility could not be predicted with CFZ, CRO, or CTX due to high major error rates.

**Conclusion:**

This study suggests potential utility of parenteral first- and third-generation cephalosporins as surrogates for oral counterparts within their respective second- and third- generations. Use of a lower breakpoint for third generation cephalosporins strengthens their role as surrogates. Future investigation is warranted to clinically corroborate these findings.

**Disclosures:**

**Kyle Molina, PharmD**, Innoviva Speciality Therapeutics: Advisor/Consultant|Melinta: Advisor/Consultant|Melinta: Grant/Research Support|Merck: Grant/Research Support|Shionogi: Advisor/Consultant

